# Supplementary material for: The interactome of intact mitochondria by cross-linking mass spectrometry provides evidence for coexisting respiratory supercomplexes
Source: Mol Cell Proteomics. 2017 Dec 8;17(2):216–32. doi: 10.1074/mcp.RA117.000470 (PMC5795388; doi:10.1074/mcp.RA117.000470)
Supplement: Supplemental Data [file supp_RA117.000470_133922_0_supp_23978_fzffwf.zip › spectra_annotation/mito_DR_spectra_annotation/499-1-4-1-12-1.pdf]

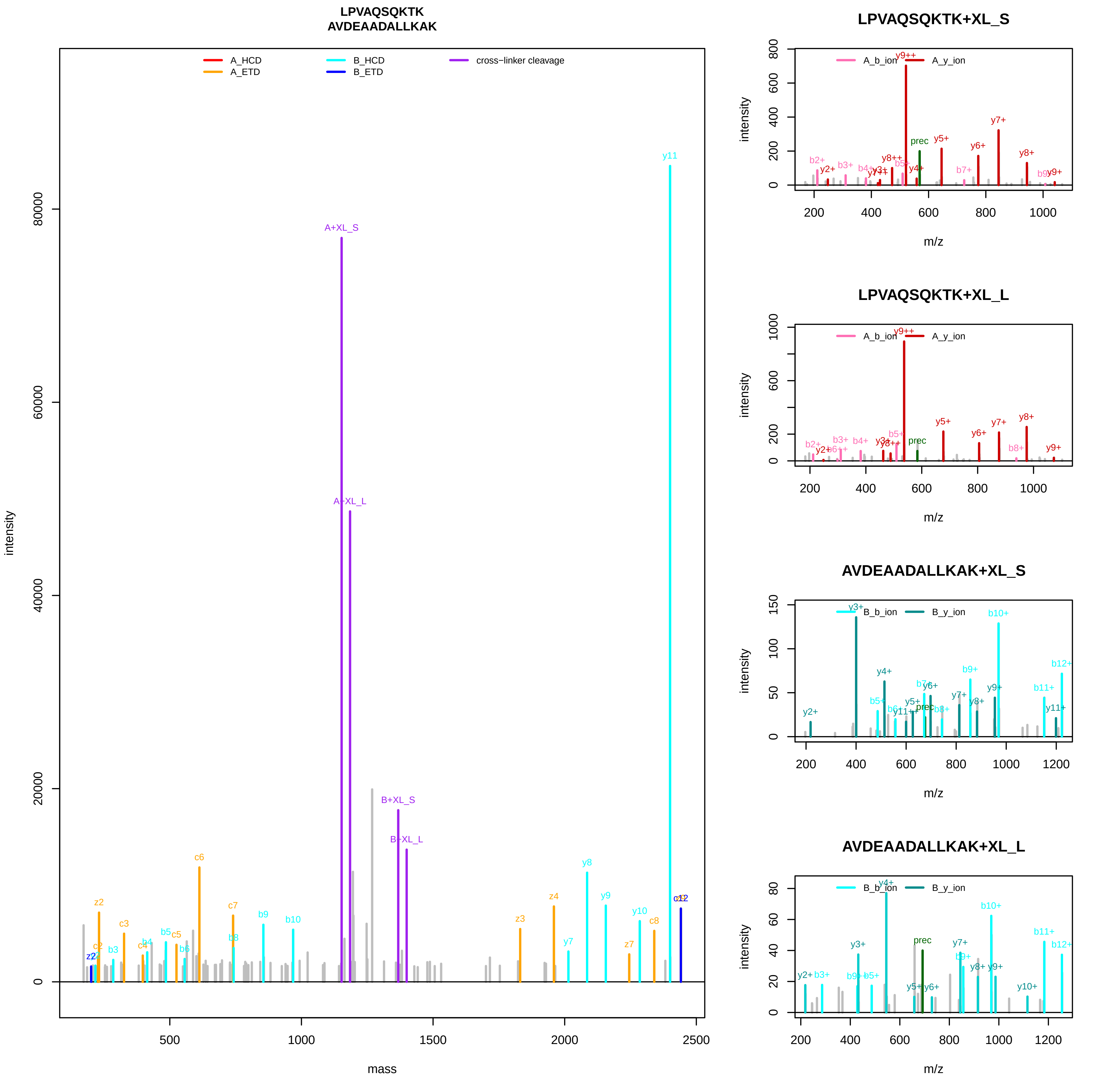
LPVAQSQKTK
AVDEAADALLKAK
A_HCD
A_ETD
B_HCD
B_ETD
cross−linker cleavage
intensity
mass
0
20000
40000
60000
80000
500
1000
1500
2000
2500
A+XL_S
A+XL_L
B+XL_S
B+XL_L
y11
y8
y9
y10
y7
z2
c2
z2
b3
c3
c4
b4
b5
c5
b6
c6
c7
b8
b9
b10
z3
z4
z7
c8
c12
LPVAQSQKTK+XL_S
LPVAQSQKTK+XL_L
AVDEAADALLKAK+XL_S
AVDEAADALLKAK+XL_L
A_b_ion
A_y_ion
B_b_ion
B_y_ion
prec
m/z
intensity
